# Preoperative ultrasound to map the three-dimensional anatomical distribution of the lateral femoral cutaneous nerve in direct anterior approach for total hip arthroplasty

**DOI:** 10.1186/s13018-021-02763-1

**Published:** 2021-10-18

**Authors:** Yu Zhang, Yao Yao, Yexian Wang, Zaikai Zhuang, Ying Shen, Qing Jiang, Dongyang Chen

**Affiliations:** 1 State Key Laboratory of Pharmaceutical Biotechnology, Division of Sports Medicine and Adult Reconstructive Surgery, Department of Orthopedic Surgery, Nanjing Drum Tower Hospital, The Affiliated Hospital of Nanjing University Medical School, 321 Zhongshan Road, Nanjing, 210008 Jiangsu People’s Republic of China; 2 Branch of National Clinical Research Center for Orthopedics, Sports Medicine Rehabilitation, Nanjing, People’s Republic of China

**Keywords:** Ultrasound, Lateral femoral cutaneous nerve, Direct anterior approach, Total hip arthroplasty

## Abstract

**Background:**

The postoperative complaints of hypoesthesia or a burning sensation due to lateral femoral cutaneous nerve (LFCN) injury in patients are not yet solved. The present study aimed to identify the three-dimensional (3D) distribution of LFCN using preoperative ultrasound and evaluate the rate of injury in direct anterior approach for total hip arthroplasty.

**Methods:**

A total of 59 patients (28 males and 31 females, age 69.0 ± 4.6 years, BMI 24.7 ± 3.0 kg/m^2^) were randomly allocated to the ultrasound group and 58 patients (28 males and 30 females, age 68.5 ± 4.5 years, BMI 24.8 ± 2.8 kg/m^2^) were in the control group. Surgeons received the data of 3D distribution of LFCN only in the ultrasound group before surgery with respect to the direction, the depth on the skin, and the length to tensor fasciae latae (TFL). The anatomical characteristics of LFCN in the surgical region were summarized. At 1 and 3 months of post surgery, the rate of LFCN injury and abnormal sensitive area was evaluated in both groups.

**Results:**

There was a significant consistency in gender, age and BMI of these two groups (*P* > 0.05). Based on the data from the ultrasound group, over 90% of patients had one or two branches of LFCN. LFCN always courses in the fascia layer, the depth ranged from 6.8 ± 2.6 (3.0–12.0) mm to 11.1 ± 3.4 (4.0–17.0) mm and depended on the thickness of the subcutaneous fat, and length was 3.3 ± 4.6 (− 5.0–10.0) mm at proximal part and − 2.7 ± 4.7 (− 10.0–8.0) at distal end to the medial edge of TFL. Both the rate of LFCN injury and abnormal sensory area in the ultrasound group was significantly lower than those in the control group (3.4% vs. 25.9%, *P* = 0.001, at 1 month; 3.4% vs. 22.4%, *P* = 0.005, at 3 months).

**Conclusions:**

LFCN mostly courses along the medial border of TFL in the fascia layer. The 3D distribution of LFCN using preoperative ultrasound mapping could help the surgeons to evaluate the risk of injury preoperatively and decrease the rate of injury during the operation. However, some branch injuries, especially for the fan type LFCN, could not be avoided.

## Background

The direct anterior approach (DAA) is increasingly preferred by surgeons when patients accept hemiarthroplasty (HTA) or total hip arthroplasty (THA) [1, 2]. This preference could be attributed to the reasons that DAA is a minimal soft invasion approach and uncovers the joint capsule through inter-muscular and inter-nervous plane [3, 4]. Compared to other approaches, such as direct lateral, anterolateral and posterior approach, patients after DAA THA had improved early ambulation capacity, fewer reoperations, enhanced functional recovery and a low dislocation rate [5–9].

However, a patient’s anxiety is due to lateral femoral cutaneous nerve (LFCN) injury that results in hypesthesia, dysesthesia or pain in the anterolateral aspect of the thigh [10–12]. This is the main complaint of the patients after DAA and causes a low satisfaction rate despite the high score of the hip function. Some studies from the clinic or cadaveric hips reported that the rate of LFCN injury was 3.29%-81.00% [10, 11, 13, 14]. This huge gap between different studies may be partially caused by surgical technique, including nerve stretching, compression, laceration, and suturing; however, the high anatomical variant rate of LFCN distribution and femoral offset, was at a higher risk for surgical injury [15–17]. The LFCN, derived from the lumbar nerve 2–3, crosses the iliacus obliquely, and then runs toward the anterior superior iliac spine (ASIS). After passing ASIS and piercing inguinal ligament, the 2 to 4 branches innervate the anterolateral aspect of the respective thigh. About 62% of the branches entered the proximal aspect of the thigh medial to the ASIS and 38% entered just above or lateral to the ASIS [17]. In addition, the fan-type branching pattern has been reported and cannot be avoided in DAA approach to the hip joint, and the LFCN injury rate of 90% in the fan-type group was significantly higher than 28.6% of in the non-fan-type group [18]. Therefore, finding a simple and efficient method to help surgeons avoid injuring LFCN, especially in patients with distribution variation, is critical.

Many studies have reported that damage to LFCN can be avoided by the preoperative identification of its distribution using ultrasound, which has higher sensitivity and equal specificity to magnetic response imaging (MRI) in noninvasive peripheral nerve visualization [16, 19–21]. Preoperative ultrasound maps the distribution of LFCN in the skin and the distal incision. However, the position of LFCN marking on the skin is easily changed with the skin in different positions of the hip joint, especially in the elderly with loose and wrinkled skin. Thus, it is not a good decision to only use skin mapping of LFCN as the reference. Herein, the three-dimensional (3D) anatomical distribution of LFCN, using ultrasound is employed to locate its position relative to the skin and muscles. We hypothesized that preoperative ultrasound identified the 3D anatomical distribution of LFCN, which decreases the rate of injury and summarizes the distribution to help the surgeons avoid LFCN during the operation.

## Methods

A total of 120 patients were randomly assigned to the control and ultrasound groups, and underwent DAA THA from September 2019 to June 2020. All patients provided informed consent, and the protocol was approved by our Faculty of Medicine-Institutional Ethics Review Board (ref. IRB 2021-413-01). Inclusion criteria: patients suffered from a fracture of neck of femur, femoral head necrosis, or osteoarthritis of the hip joint and need primary hip arthroplasty. Exclusion criteria: patients had a poor cardiopulmonary function, and hence, could not be burdened with the crisis of anesthesia and surgery and experienced other operations in the same hip joint with scar or anatomical disorder; the patients with a history of neurological abnormalities in the thigh were excluded. Moreover, general characteristics, including age, sex, and body mass index (BMI), were evaluated.

### Ultrasound mapping

**Fig. 1:**
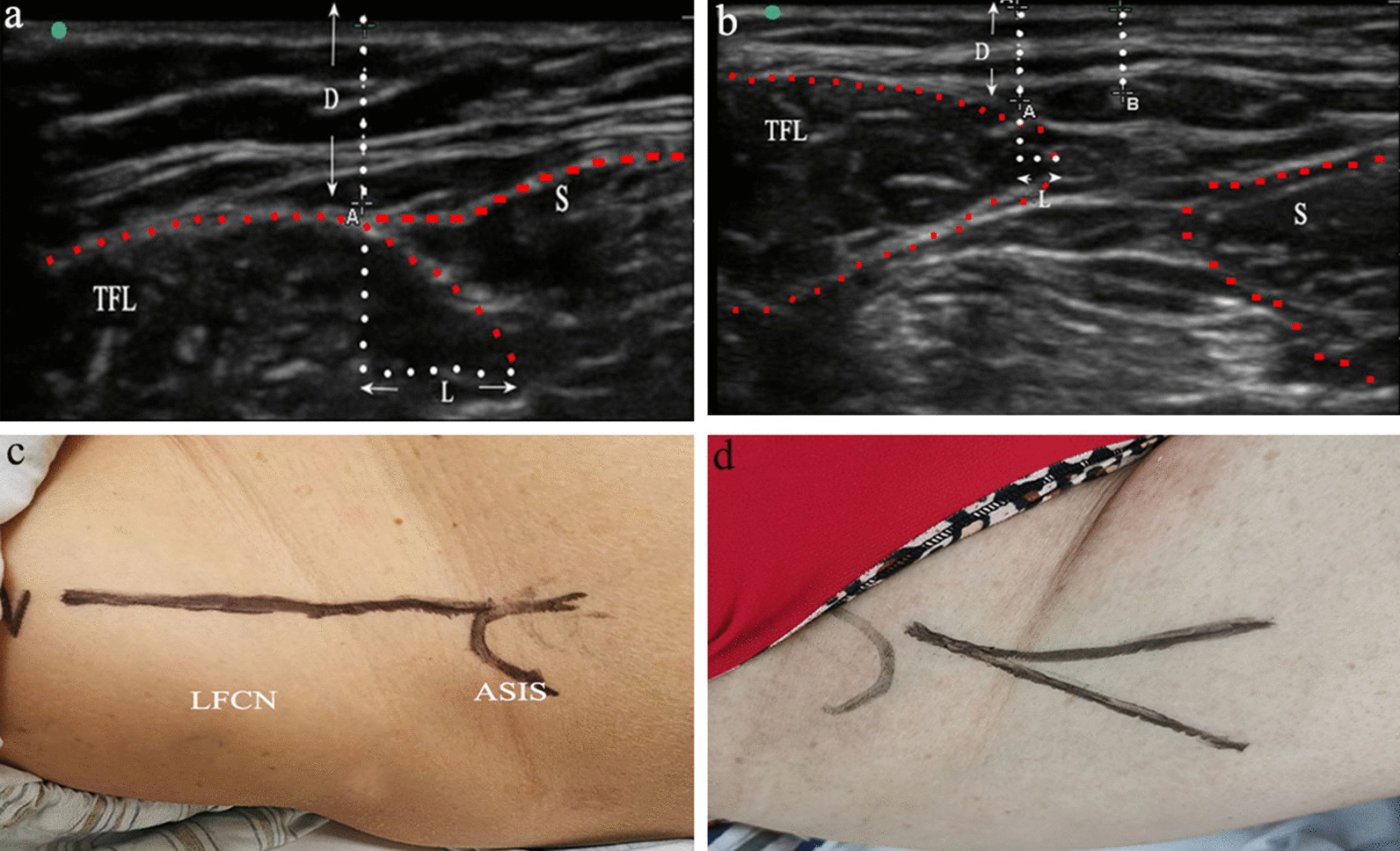
3D identification of LFCN with Doppler ultrasound photograph

In the ultrasound group, two skilled physiatrists with neuromuscular ultrasound experience examined the 3D distribution using an ultrasound machine (SonoSite M-turbo, USA) with a 10 MHz linear array transducer to detect the stem of LFCN at beginning. Then, the superior small branches would be detected via changing the frequencies probe based on the depth of LFCN stem. The ASIS and sartorius muscle was used as a reference to describe the continuous course of LFCN. Three cross-sectional areas were selected to trace the position of LFCN relative to skin and tensor fasciae latae (TFL). The first point was that LFCN left the pelvis near ASIS and inguinal ligament (IL), and the second and third recording points were 5 cm and 10 cm distal to the ASIS, respectively. The depths of LFCN to the skin at the three points were recorded as D1, D 2, and D3, respectively. The lengths of LFCN to the medial edge of TFL at the three points were recorded as L1, L 2, and L3, respectively. If LFCN was located on the medial side of TFL, it was recorded as –L, and if lateral, it was + L (Fig. 1a, b). Finally, the course of the LFCN was mapped on the skin using a marker pen. These data will be taken as reference for surgeons to avoid damaging LFCN during operation (Fig. 1c, d). In the control group, nothing tracked the course of LFCN.

### Surgical technique

All surgeries were performed by one experienced DAA surgeon and three assistant surgeons. After intravenous anesthesia, the participant lied on a standard surgical bed in the supine position, and the hip joint that required arthroplasty was 10 cm higher than the other side. The skin incision was 2 cm lateral from ASIS and proceeded distally for about 8–10 cm that was parallel to the line of ASIS and lateral of the patella. Briefly, after subcutaneous and fascia dissection, the sartorius, temoriss, and tensor fascia were isolated, the muscle interval was uncovered, and the branches of lateral femoral circumflex artery ligated. The anterior capsulotomy and femoral neck osteotomy were performed, and then the acetabulum was exposed. The acetabulum was prepared using different sizes of offset reamers at 40–45° abduction and 15° anteversion until the surface oozed blood. After the installation of an artificial prosthesis of the acetabulum, the femoral preparation continued in a modified figure-four position of the leg with 45° hyperextension and elevation of the femur by a double-tipped retractor behind the greater trochanter. The distance from the horizontal line of the great trochanter to the center of the rotation of the femoral head based on the result of pelvic radiograph was used to evaluate the length of the leg, and the range of movement was effectuated to assess the stability of leg. After introducing the artificial femoral component and head, the deep fascia, subcutaneous tissue, and intracutaneous were sutured step by step.

**Fig. 2:**
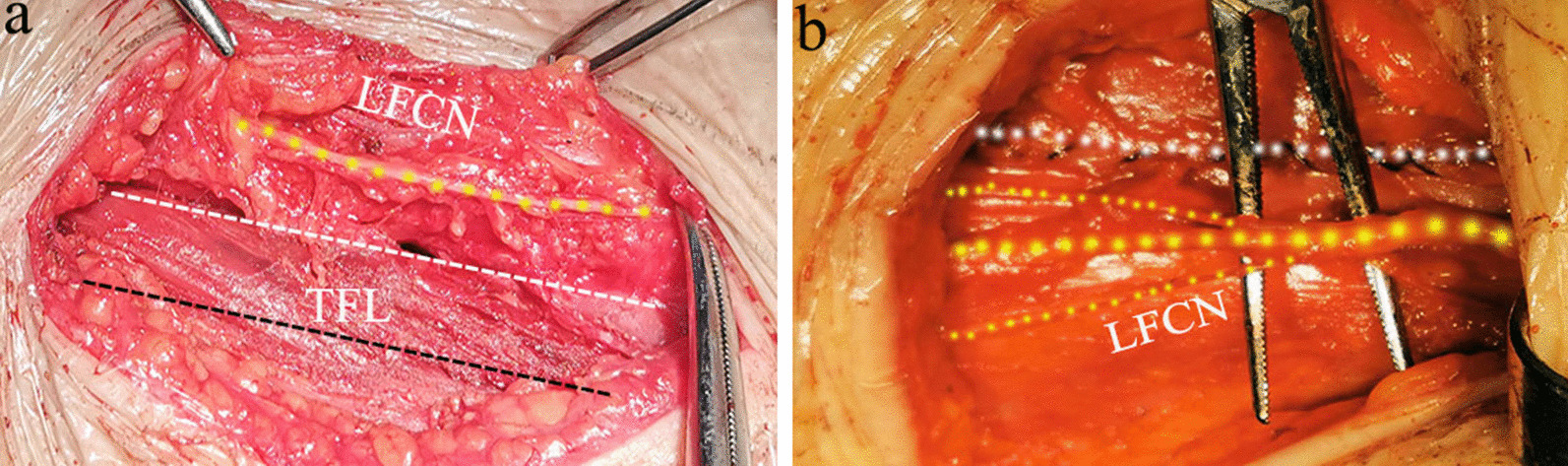
The intraoperative anatomy of LFCN showed its relative location relationships between incision and TFL

To identify the consistent results between preoperative ultrasound data and anatomical positions, five patients were informed and consented to expose the LFCN in surgery. Based on the preoperative ultrasound 3D distribution data, including the depth to skin, the mapping path and the length to TFL, we accomplished to show the anatomy of the LFCN intraoperation. It demonstrated the nerve located medical side of our incision and provided practical parameters for surgeons to keep the suture at an appropriate distance from border of incision while avoiding the LFCN (Fig. 2).

### Evaluation of LFCN injury in patients

**Fig. 3:**
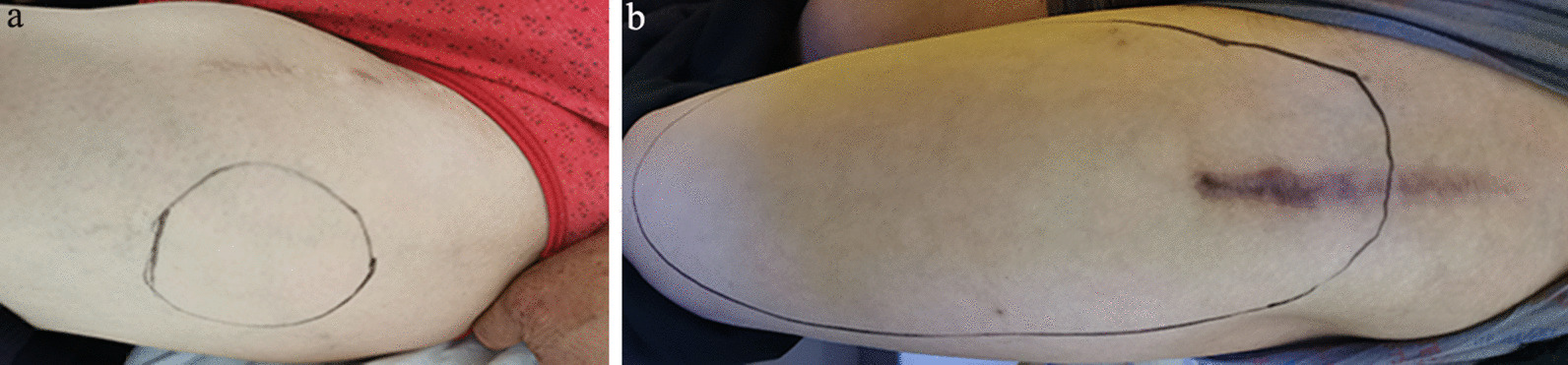
Sensory abnormal area after surgery

The operation time was calculated and compared between ultrasound group and control group because that the longer time was used to pull and expose surgical field and the more possible to injury nerve. At 1 month and 3 months after surgery, all patients underwent follow-up in the Outpatient Department. Also, the abnormal sensation in the anterolateral thigh, including hypesthesia, dysesthesia, numbness, and pain was assessed. If a patient experiences abnormal sensation, he/she would be asked to mark the region area using a black marking pen (Fig. 3). This area was estimated by multiplying the longest diameter with the shortest diameter. An experienced doctor assessed the function of hip joint after DAA THA surgery.

### Statistics

As the continuous variables are presented as mean ± SD, Student’s *t* test was used to analyze data for normal distribution and Mann–Whitney *U* test for abnormal distribution. On categorical variables, Fisher’s exact test was used. *P* < 0.05 indicated the statistical significance. It was calculated that 45 patients were required in each group to detect a difference in the rate of LFCN injury with a standard deviation of 14.8, 90% power and a two-sided alpha error of 0.05. Statistical Package for the Social Science version 23.0 (INM Corporation, Armonk, NY, USA) was utilized to analyze these data.

## Results

### Demographics, baseline patient information, and the 3D distribution of LFCN

A total of 117 patients completed the follow-up at 1 month and 3 months after operation. The ultrasound group had 59 patients (28 men and 31 women; average age, 69.0 ± 4.6 years (58–79 years)), while the control group had 58 patients (28 men and 30 women; average age, 68.5 ± 4.5 years (60–79 years)). Of these, 28 (23.9%) patients suffered from femoral neck fracture (FNF), 54 (46.2%) from femoral head necrosis (FHN), and 35 (29.9%) had osteoarthritis (OA). The mean BMI was 24.7 ± 3.0 (17.2–31.5) kg/m^2^ and 24.8 ± 2.8 (18.5–31.2) in the ultrasound and control groups, respectively (Table 1). In the ultrasound group, 30 (54.5%) of LFCN were identified one branch, 23 (41.8%) had two branches, and 2 (3.7%) had at least three branches in the surgical region. The distance from the skin surface to LFCN was termed as D1, D2, and D3. The results of D1, D2, and D3 were 6.8 ± 2.6 (3.0–12.0) mm, 9.2 ± 2.8 (3.0–15.0) mm, and 11.1 ± 3.4 (4.0–16.0) mm, respectively. The LFCN courses in the fascia that is on the surface of the sartorius and TLF within 10 cm from the far end of ASIS. The length from LFCN to the medial edge of TLF named L1, L2, and L3 was 3.3 ± 4.6 (− 5.0–10.0) mm, 0 ± 4.1 (− 10.0–7.0) mm, and − 2.7 ± 4.7 (− 10.0–8.0) mm, respectively (Table 2).

**Table.1:** Demographic and medical history in patients with and without ultrasound definition of LFCN

	Ultrasound group	Control group	*P*	*t/χ*^*2*^
*Sex*				
Male	28 (47.5%)	28 (48.3%)	1.000	*χ*^2^ = 0.008
Female	31 (52.5%)	30 (51.7%)		
Age (years)	69.0 ± 4.6 (58–79)	68.5 ± 4.5 (60–79)	0.608	*t* = 0.514
BMI (Kg/m^2^)	24.7 ± 3.0 (17.2–31.5)	24.8 ± 2.8 (18.5–31.2)	0.827	*t* = 0.219
*Disease*				
FNF	15 (25.4%)	13 (22.4%)	0.163	*χ*^2^ = 0.922
FHN	27 (45.8%)	27 (46.6%)		
OA	17 (28.8%)	18 (31.0%)		

LFCN, the lateral femoral cutaneous nerve; BMI, body mass index; FNF, femoral neck fracture; FHN, femoral head necrosis; OA, osteoarthritis

**Table.2:** *D* from skin and *L* from the medical edge of TFL

	*D* (mm)	*L* (mm)
1	6.8 ± 2.6 (3.0–12.0)	3.3 ± 4.6 (− 5.0–10.0)
2	9.2 ± 2.8 (3.0–15.0)	0 ± 4.1 (− 10.0–7.0.0)
3	11.1 ± 3.4 (4.0–17.0)	− 2.7 ± 4.7 (− 10.0–8.0)

D, depth; L, length

### The post-surgery function of the hip joint and the injury of LFCN

Before surgery, the preoperative hip Harris scores were assessed in these two groups, and patients with femoral neck fracture got 0 because they were too painful to perform any hip joint action and needed to stay in bed. The patients in the ultrasound group got 22.9 ± 14.7, and the control group was 24.2 ± 15.1 (*P* = 0.640 > 0.05, *t* = 0.468), all patients had poor hip joint function preoperatively (the Harris score < 70). At 1 month and 3 months, the hip Harris scores were 79.6 ± 3.5 (72–85) and 89.6 ± 3.4 (82–95) for the ultrasound group and, 80.0 ± 3.7 (72–86) and 90.0 ± 3.8 (84–96) for the control group, respectively. No significant difference was detected between the two groups. The time of operation was also no significant difference between these two groups, which indicated that the ultrasound identification of the LFCN was accomplished before operation and did not dramastically impact the surgical procedure and treatment effect. Also, no participant suffered from infection, poor wound healing, prosthetic loosening, or fracture around the prosthesis.

At 1 month after the operation, 2 (3.6%) patients reported numbness or dull sensation in the cutaneous area of the anterolateral thigh in the ultrasound group, and the symptoms area was 33.0 ± 10.8 (21.0–42.0) cm^2^, and the region was located in the lateral area of the incision. The LFCN of the two patients had three branches at the end, and the lateral branch passed the TFL. In the control group, 15 (24.2%) patients described abnormal symptoms in the anterolateral thigh, including numbness (15), dull sensation (15), and tingling or pain (4) in the 1st month follow-up, which was significantly higher than that in the ultrasound group. The area of abnormal symptoms of the control group was 133.1 ± 104.9 (49–375) cm^2^, which was significant larger than that of ultrasound guiding group. At 3 months, both the number of patients and the area of abnormal symptoms did not show any obvious change in the ultrasound group. In the control group, the tingling or pain disappeared completely at 3 months; in 2/4 patients suffering from tingling or pain, the normal sensation was restored. Also, the area of numbness or dull sensation did not show a significant difference as compared to that at 1 month after the operation, while the area of abnormal symptoms of the control group was 132.7 ± 112.9 (49–375.0) cm^2^ (Table 3).

**Table.3:** Rate of LFCN injury, sensory disturbance area, and Harris scores at 1 and 3 months after operation in the ultrasound and control groups

	Ultrasound group			Control group			*P*_*preop*_ *t*_*preop*_	*P*_1_ *t*_1_**/***χ*^2^_1_	*P*_3_ *t*_3_**/***χ*^2^_3_
	Preop	1 month	3 months	Preop	1 month	3 months			
LFCN injury	0	2 (3.4%)	2 (3.4%)	0	15 (25.9%)	13 (22.4%)	–	0.001 *χ*^2^_1_ = 11.893	0.005 *χ*^2^_3_ = 9.471
Area (cm^2^)	0	39.0 ± 4.2 (36.0–42.0)	39.0 ± 4.2 (36.0–42.0)	0	133.1 ± 104.9 (49.0–375.0)	132.7 ± 112.9 (49–375.0)	–	< 0.001 *t*_1_ = 4.048	0.002 *t*_3_ = 4.139
Harris score	22.9 ± 14.7 (0–48)	79.6 ± 3.5 (72–85)	89.6 ± 3.4 (82–95)	24.2 ± 15.1 (0–51)	80.0 ± 3.7 (72–86)	90.0 ± 3.8 (84–96)	0.640 0.468	0.517 *t*_1_ = 0.651	0.518 *t*_3_ = 0.649
Time of operation		84.8 ± 5.5			82.2 ± 5.2			0.638 *t* = 2.670	

LFCN, the lateral femoral cutaneous nerve

## Discussion

As a mini-invasion approach, DAA for THA is increasingly popular in the clinic because of soft tissue preservation using the inter-muscular and nervous plane, allowing for more reproducible and precise cup placement in supine posion, and fast functional discovery after surgery [9, 22]. However, the abnormal sensation caused by LFCN injury on the area of anterolateral thigh, including numbness, paresthesia, and pain, is the most common complaint of patients after surgery [14, 23]. The main reasons that result in LFCN injury include two aspects: ① the anatomical variations and frequent branches of LFCN; ② the DAA approach close to the path of LFCN. In the proximal femur, LFCN courses the intermuscular space between the TFL and sartorius muscles, which is also the surgical field [17, 24, 25]. These two factors increase the risk of cutting or suturing of LFCN interoperation, which are the main reasons for LFCN injury.

Ultrasound is the first choice level technique to image the nerve according to the last Guidelines of the European Society of Musculoskeletal Radiology (ESSR) [26] and widely applicated in ultrasound-guided injections around the nerve [27, 28], we attempted to use ultrasound to identify the 3D distribution, which would help to avoid damaging LFCN from cutting to the suture. This is different from traditional skin marking protocol using ultrasound guidance. Skin marking follows the skin movement especially in elderly or patients with loose skin, which causes uncertain localization of LFCN on the skin surface. The 3D distribution identification provides three types of data to the surgeons, including path, depth, and distance to TFL in the surgical region. Subsequently, the D was ≤ 2 cm from the point of LFCN exit (between the medial of ASIS and lateral 1/3rd of the inguinal ligament) to the ASIS. The depth of LFCN to the skin was deeper in the far end than that in the exit location, and this phenomenon was obvious in the obese patients who had thick subcutaneous fat. In the surgical region, LFCN was a single branch in the exit point, and always in the fascia latae with a bilayer structure. In the distal end of ASIS within 10 cm, LFCN mainly had a single branch, followed by two types of branches and then, three or more branch types. The more branches the LFCN had, the closer to TFL was, and the higher risk of LFCN injury. Three or more branches of LFCN were collectively termed as fan-type. In this study, two patients were identified fan-type, and the lateral branch of LFCN of 3 patients was cut as it obstructed the operation exposure. Another study had been reported that LFCN named as the fan-type injury cannot be avoided in DAA surgical dissections [18]. Herein, it was also demonstrated that the surgeon had to cut some branches of LFCN to acquire adequate surgical vision field though found them in patients with fan-type LFCN. The average distance to medical TFL was about 15 mm, and it became closer to TFL from proximal to distal. If there is more than one branch, the lateral branch of LFCN passes the lateral side of TFL, indicating that the incision of DAA should be located at least 10 mm distance from the medical side of TFL. The skin was marked with the line of the medical side of TFL was the line of ASIS and lateral condyle of the femur. In addition, to avoid the suturing injury of LFCN and result in meralgia paresthetica, another 5 mm distance from LFCN to incision should be added for the suture fascia layer. Therefore, the DAA incision needs to be localized15mm to the lateral side of the line of ASIS and lateral condyle of the femur.

The 3D location of LFCN using non-invasiveness of ultrasonography, which was not just a traditionally projection onto the skin [29], provided multidimensional distribution information for surgeons, with respect to the LFCN during incision of the skin and opening and closing of the fascia layer. Compared with no 3D location, the identification of LFCN was a great option to significantly decrease the rate of DAA-induced LFCN injury. Here, 10 MHz transducer was used to detect the stem of LFCN firstly, and then higher frequencies probe was used to avoid omitting some superficial branches because that different frequencies provided different sensitivities in various depths [30]. Similar studies, which focused on the treatment of meralgia paresthetica of LFCN or nerve block conduction using ultrasound guiding technique, also demonstrated that ultrasonographic images provided precious and visible position that would be helpful to a safe and effective treatment, especially for nerves with anatomical variation and some branches [31–33].

In addition, almost the patients who suffered from LFCN injury in the control group complained of troubled paresthesia in first follow-up. After patients were explained and made comfortable, they could understand and accept the situation. Therefore, the identification of LFCN before surgery was also an optimal option for patients, especially for those with the fan-type LFCN. Based on the location of DAA incision and LFCN distribution, the surgeons could easily evaluate the risk level of LFCN injury during the operation and explain to the patients that the branches of LFCN will be injured and some symptoms of sensory disturbance will be observed after the operation. Thus, the patients will accept the situation and let their mind at ease, which would alleviate the conflicts between doctors and patients.

Nevertheless, the method of identification of LFCN using ultrasound also has some limitations. It needs an experienced ultrasonography doctor to search for LFCN due to its variation. The ultrasound identification examination also adds to the cost of inpatient. Also, additional time and energy would be needed to record the map of LFCN on the skin and some parameters due to individual variation. In this study, we identified the location of LFCN preoperatively but did not identify whether it was intact or injured postoperatively. The chief complaint of the patients was the only evidence to evaluate the situation of LFCN in the follow-up. Therefore, the rate of injury in LFCN may be influenced by the subjective factor of patients. In addition, as for age or BMI, there was no significant difference between these two groups, but age or BMI were possible risk factors of damaging the LFCN because young patients with strong muscles and obese patient need strong force to pull their incisions to obtain good surgical vision. And, pulling during operation was a risk to increase the rate of nerve injury.

## Conclusions

Before surgery, it is a choice for the surgeons for DAA-THA to identify the 3D distribution of LFCN using ultrasound. Based on the marking on the skin and location to TFL, surgeons can make a preoperation plane and select an incision to reduce the rate of LFCN injury, or even inform the patients before surgery that some branches of LFCN will be cut during the operation. This would alleviate their anxiety and the conflicts between the doctors and patients postoperatively. In addition, the anatomical parameters from the ultrasound group also provided a general reference for doctors who could not implement ultrasound-guided identification of LFCN for every patient who underwent DAA-THA.

## Data Availability

The datasets used and/or analyzed during the current study are available from the corresponding author on reasonable request.

## Abbreviations

LFCN: Lateral femoral cutaneous nerve
DAA: Direct anterior approach
THA: Total hip arthroplasty
3D: Three-dimensional
TFL: Tensor fasciae latae
ASIS: Anterior superior iliac spine
MRI: Magnetic response image
IL: Inguinal ligament
FNF: Femoral neck fracture
FHN: Femoral head necrosis
OA: Osteoarthritis
BMI: Body mass index
